# Wall motion recovery in dobutamine stress magnetic resonance imaging

**DOI:** 10.1186/1532-429X-13-S1-P99

**Published:** 2011-02-02

**Authors:** Alexander Berger, Sebastian U Kelle, Christopher Schneeweis, Michael Frick, Christoph Klein, Eckart Fleck, Rolf Gebker

**Affiliations:** 1 German Heart Institute Berlin, Berlin, Germany

## Objectives

To correlate the persistence of new or worsening induced wall motion abnormalities in dobutamine stress magnetic resonance imaging (DSMR) and the extent of coronary artery disease (CAD) in invasive coronary angiography.

## Background

DSMR is a highly sensitive and specific non-invasive method for detecting induced wall motion abnormalities (IWMAs) in patients with significant CAD. Yet little is known about the duration of IWMA during the recovery period. We hypothesize that the persistence of IWMAs during recovery may be associated with the extent of CAD.

## Methods

**Figure 1:**
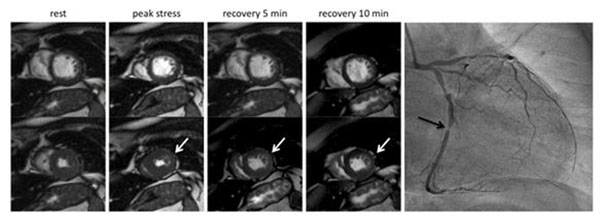
Left side: Short axis views during diastole (top row) and systole (bottom row) showing wall motion at rest, at peak stress level, after five minutes recovery and 10 minutes recovery. The white arrows mark the induced wall motion stress abnormality which sustains over the recovery period of ten minutes. Right side: Coronary angiography of the same patient showing a high grade stenosis of the mid RCX (black arrow).

DSMR was performed in twenty-eight consecutive patients with suspected or known CAD scheduled for clinically indicated invasive coronary angiography. Each patient underwent routine DSMR including cine imaging at rest and during dobutamine infusion. Additionally, three standard short axis and longitudinal axis views were obtained after five, ten and fifteen minutes during the recovery period in order to detect persisting wall motion abnormalities in ischemic myocardial territories, which were assigned to coronary arteries based on the 17 segment model. Intermediate and severe coronary stenosis were defined as 50-75% and >75% luminal narrowing using invasive coronary angiography, respectively (figure 1). All patients received esmolol after stopping the dobutamine infusion to achieve a heart rate under 100bpm.

## Results

**Figure 2:**
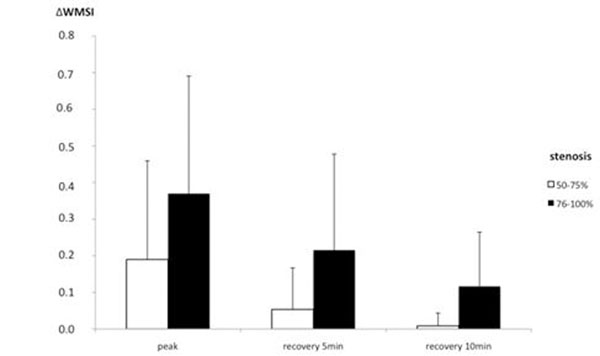
Significant difference of the Δ wall motion score index (ΔWMSI) at peak stress level (p=0.03), during recovery phase at 5 minutes (p=0.007) and 10 minutes (p=0.005) comparing intermediate and severe coronary artery stenosis. The higher the wall motion score index was at peak stress, the longer IWMAs persisted during the recovery phase (p<0.01).

The recovery time was associated with more severe CAD. Our data showed that the higher the wall motion score index was at peak stress, the longer IWMAs persisted during the recovery phase (p < 0.01, see figure 2).

The wall motion score index showed significant differences in territories supplied by arteries with intermediate stenoses compared with severe stenoses under maximum stress (difference wall motion score index ΔWMSI 0.19±0.27 vs. 0.37±0.32, p=0.03) and at five (ΔWMSI 0.05±0.11 vs. 0.22±0.26, p=0.007) and ten minutes (ΔWMSI 0.01±0.04 vs. 0.12±0.15, p=0.005) during recovery phase.

Patients with intermediate and those with severe stenoses demonstrated no significant difference in the rate pressure product at maximum stress level (p=0,5), during recovery after five minutes (p=0.38) and ten minutes (p=0.16).

## Conclusions

We demonstrated that normalization of left ventricular IWMAs is related to the extent of CAD. Cine imaging during the recovery phase may be helpful for additional risk stratification.

